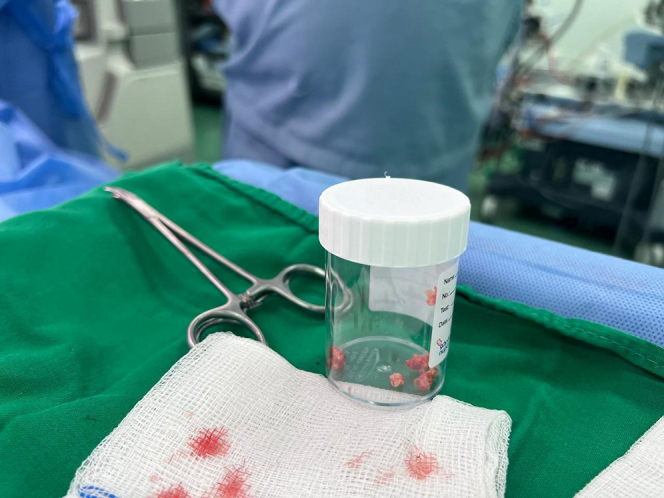# Methicillin-Resistant *Staphylococcus aureus* Aortic Valve Endocarditis With Cerebral and Peripheral Artery Embolization

**DOI:** 10.1016/j.atssr.2025.12.014

**Published:** 2026-01-10

**Authors:** Hassane Abdallah, Sultan Askar, Anwar Al Hulaibi, Mohannad Dawary, Fatemah Al-Aithan, Jaafer Al-Obaid, Mohammed Abdulkader, Mohammed Al Hemmyari, Haitham Altaani

**Affiliations:** 1 Department of Cardiac Surgery, Prince Sultan Cardiac Center–Al Hassa, Saudi Arabia; 2 Department of Cardiac Surgery, Saud Al Babtain Cardiac Center, Dammam, Saudi Arabia; 3 Department of Cardiac Surgery, King Faisal Specialist Hospital & Research Center, Riyadh, Saudi Arabia; 4 Department of Radiology, King Fahd Hospital–Al Hassa, Saudi Arabia; 5 Department of Perfusion, Prince Sultan Cardiac Center–Al Hassa, Saudi Arabia; 6 Department of Anesthesia, Prince Sultan Cardiac Center–Al Hassa, Saudi Arabia

## Abstract

Infective endocarditis can affect native valves, prosthetic valves, or implanted cardiac devices. It has a high in-hospital mortality rate. This is a 43-year-old woman with a history of aortic valve replacement 4 months before presentation. She presented with fatigue. Computed tomography showed left internal carotid artery occlusion and bilateral cerebral infarcts. Echocardiography showed a dehiscent prosthetic valve, aortic root pseudoaneurysm, and abscess. She underwent redo aortic valve replacement with root reconstruction. Infective endocarditis with carotid artery disease is a complex condition. Septic emboli can affect the carotid arteries, leading to stroke. Early detection and a comprehensive examination are required to optimize outcomes.

The Supplemental Material can be viewed in the online version of this article [https://doi.org/10.1016//j.atssr. 2025.12.014] on https://www.annalsthoracicsurgeryshortrep.org

Infective endocarditis (IE) represents a microbial infection of the endocardial surface of the native valve, a prosthetic valve, or an implanted cardiac device, with an estimated incidence ranging from 3 to 10 cases per 100,000 per year.^1^ Despite technological improvement in diagnostic and therapeutic strategies for IE, the overall in-hospital mortality remains high.^2^ A well-known complication of valve replacement is prosthetic valve endocarditis. In recent decades, there has been an increase in the overall incidence of IE secondary to *Staphylococcus aureus*, which is a devastating infection with a reported mortality rate of >40% to 80%. Moreover, it is believed that the mortality rate for methicillin-resistant *S*. *aureus* (MRSA) IE is even greater.^3^ Life-threatening embolic events may result once intracardiac vegetations are identified, in which the overall risk of peripheral embolism ranges from 20% to 50%, and most occur in the central nervous system, causing ischemic stroke.^2^ However, there are very few case reports of complete internal carotid artery occlusion in the literature, indicating how rare this condition is.^4^

A 43-year-old woman, with history of aortic valve replacement with a mechanical prosthesis due to aortic stenosis 4 months ago, presented to the emergency department complaining of fatigue. The patient was afebrile, with a heart rate of 88 beats/min with regular rhythm, blood pressure of 111/59 mm Hg, respiratory rate of 18 breaths/min, and pulse oximetry of 100% on room air. Physical examination was unremarkable. The patient was admitted for further investigation. Hours later, her Glasgow Coma Scale score dropped.

Laboratory data showed a leukocytosis of 27 mm^3^ and normal renal and liver function. Broad-spectrum antibiotics were started once blood cultures, which grew MRSA, were obtained. The patient underwent a brain computed tomography scan that revealed a right basal ganglia and multiple small bilateral infarcts as well as a left internal carotid artery occlusion (Supplemental Figure 1). Moreover, she underwent magnetic resonance imaging, which showed multiple acute and subacute infarcts in both cerebral hemispheres as well as occlusion of the left internal carotid artery due to acute thrombosis (Figure 1).

**Figure 1:**
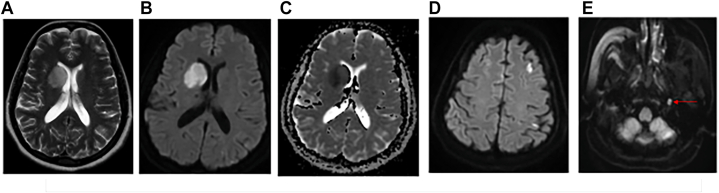
(A-C) Magnetic resonance imaging of the brain at the level of basal ganglia: (A) T2-weighted image, (B) diffusion-weighted image, and (C) apparent diffusion coefficient map show high T2 signal intensity within the right basal ganglia associated with restricted diffusion consistent with acute infarction. (D) Axial diffusion-weighted image at centrum semiovale shows similar infarct foci scatter in left cerebral hemisphere. (E) Axial diffusion-weighted image shows restricted diffusion (arrow) in left internal carotid artery, denoting occlusion.

Transesophageal echocardiography was performed, revealing a dehiscent mechanical aortic valve with large pseudoaneurysm associated with large abscess and large vegetations protruding into the left ventricular outflow tract. Furthermore, a significant paravalvular leak was identified (Figure 2; Supplemental Figure 2; Videos 1-6). After a heart team meeting involving cardiac surgery, cardiology, vascular surgery, and infectious diseases specialists, the decision was made to take the patient for an emergency redo aortic valve replacement.

**Figure 2:**
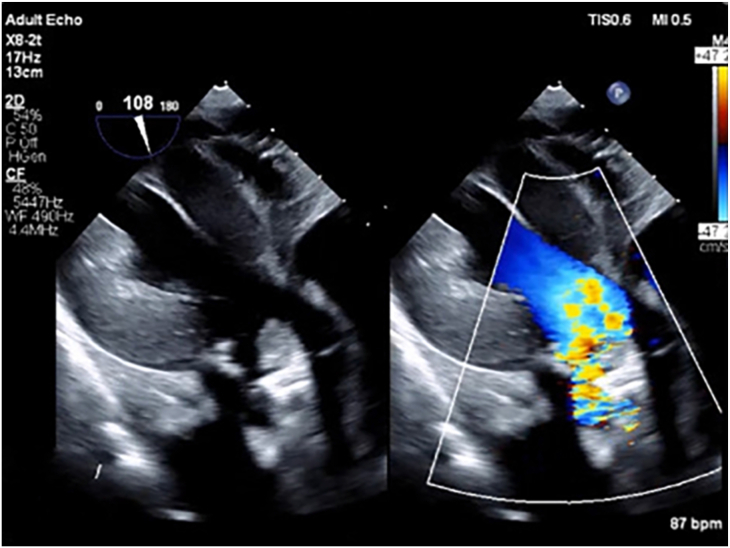
Transesophageal echocardiography image, transgastric long-axis view at 108 degrees (systolic frame), showing a large mass (vegetation) attached to the bileaflet mechanical aortic valve. In real time, color flow imaging revealed a significant paravalvular leak.

A right femoral cutdown was performed. A blue discoloration, likely representing an embolic vegetation, was identified in the right common femoral artery at the level of bifurcation. Femoral arterial cannulation was done above the level of the embolus. Next, median sternotomy was performed.

An aortic cross-clamp was applied, and diastolic arrest was achieved by retrograde cardioplegia. After the aortotomy, inspection of the valve revealed a dehiscent prosthesis; aortic root pseudoaneurysm at the level of the noncoronary cusp; destruction of the aortomitral curtain; and extension of infection/necrotic tissue to the ventricular side, the interventricular septum, and the right coronary ostia. The prosthetic valve was excised, and the aortotomy was extended to the noncoronary cusp and then to the aortomitral curtain. Radical débridement of all necrotic tissues was done. The aortomitral area was reconstructed with a bovine pericardial patch, which was extended to the aortic root (Video 7). A 21-mm mechanical valve was implanted with pledgeted sutures from both the ventricular and arterial sides. Next, the patch was extended to the aortic root and closed with aortotomy incision after air was removed. The patient also required a saphenous vein graft to the right coronary artery.

The vascular surgery service was consulted intraoperatively for the right femoral artery embolization. A femoral artery endarterectomy and a saphenous vein patch angioplasty were performed (Supplemental Figures 3 and 4).

Postoperatively, a permanent pacemaker was inserted on the eighth postoperative day for third-degree heart block. The patient completed a 6-week antibiotic course and was discharged 45 days after surgery. She presented to the clinic 3 months after discharge in a stable condition.

## Comment

Embolization of endocardial vegetations can cause an ischemic embolism in a number of places, including the mesentery, spleen, kidney, and cerebral vasculature.^5^ The overall risk of peripheral embolism ranges from 20% to 50%.^2^ Rarely, large vessels, such as the middle cerebral artery or internal carotid artery, can be occluded by emboli.^5^ Studies have shown that *S. aureus* IE is associated with higher rates of central nervous system embolic events, especially when the anterior leaflet of the mitral valve is affected.^6^ Wolf and coworkers^5^ presented a case of an internal carotid occlusion resulting from a septic embolism in the setting of mitral valve MRSA IE.

In patients with IE, use of thrombolytics is not recommended because of the increased risk of hemorrhagic consequences.^7^ A mechanical or endovascular thrombectomy may be considered if a large-vessel occlusion is confirmed in the setting of acute ischemic stroke as a result of IE.^8^ Matsukawa reported the first case of IE-related internal carotid artery occlusion treated with endovascular thrombectomy.^9^

Optimal management of carotid disease in conjunction with bacterial endocarditis and bloodstream infections is still not well established because of the limited available body of evidence. The standard approach in treating symptomatic high-grade carotid stenosis involves either placement of a carotid stent or performance of a carotid endarterectomy. However, in cases of carotid stenosis with recent or active endocarditis or bacteremia, the possibility of infected atheroma should be kept in mind; a potential source of bacteremia may be prolonged by placing a foreign body over a potentially infected plaque, such as a patch graft, during carotid endarterectomy or a stent during carotid angioplasty.

In conclusion, bacterial endocarditis with bloodstream infections alongside carotid artery disease presents a complex and potentially life-threatening clinical situation. In addition, septic emboli from infected cardiac valves may directly involve the carotid circulation, resulting in local vascular complications, such as arterial wall damage and mycotic aneurysms. Optimizing outcomes for affected individuals requires early detection; comprehensive vascular and cardiac examination; and a multidisciplinary approach that includes antimicrobial therapy, potential surgical intervention, and stroke prevention.

## Figures and Tables

**Supplemental Figure 1:**
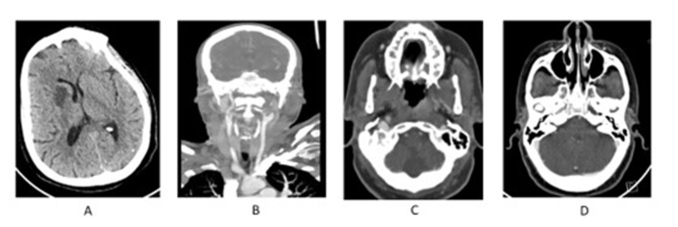


**Supplemental Figure 2:**
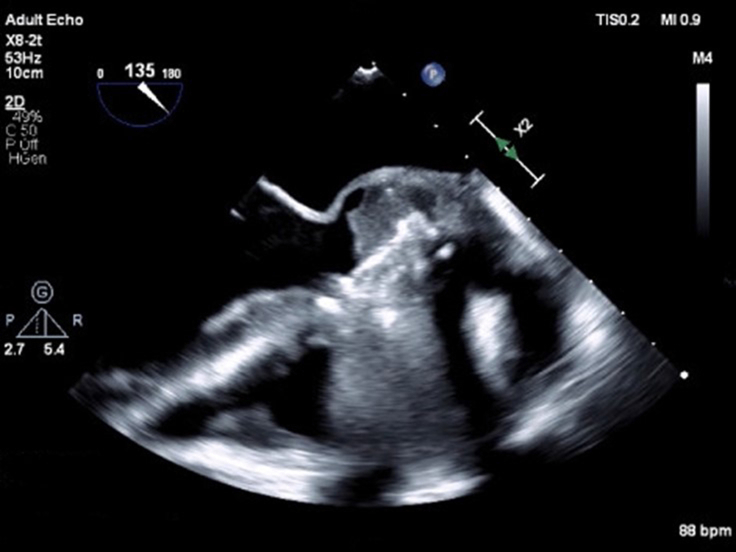


**Supplemental Figure 3:**
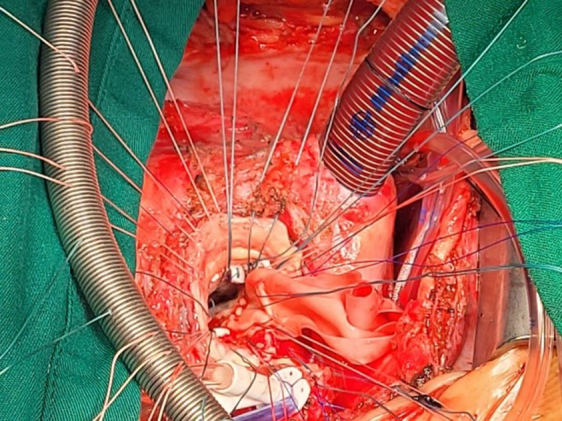


**Supplemental Figure 4a:**
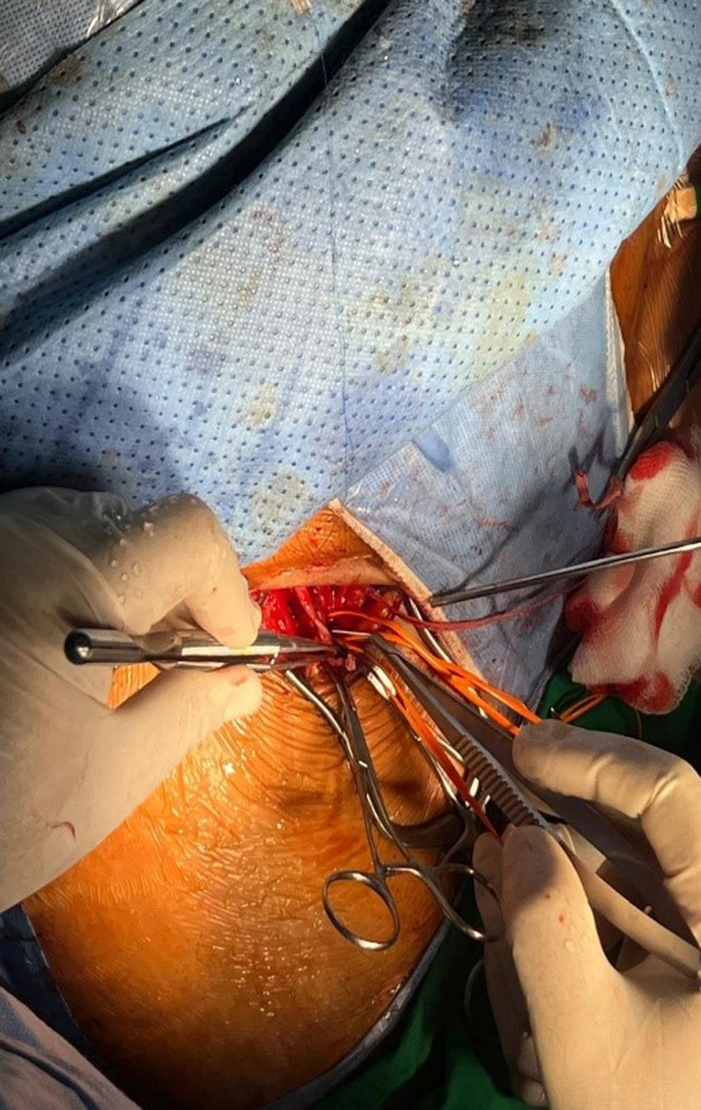


**Supplemental Figure 4b:**