# Supplementary material for: Methicillin-Resistant Staphylococcus aureus Aortic Valve Endocarditis With Cerebral and Peripheral Artery Embolization
Source: Ann Thorac Surg Short Rep. 2026 Jan 10;4(2):645–7. doi: 10.1016/j.atssr.2025.12.014 (PMC13245505; doi:10.1016/j.atssr.2025.12.014)
Supplement: Supplemental Figure and Legends [file mmc8.docx]

**Supplemental figure 1:**

Plain CT brain (A) axial cut at the level of basal ganglia shows hypodensity in the right basal ganglia, denoting acute infarct.

Cerebral angiogram (B) coronal MIP, (C &D) axial cuts show occlusion of left internal carotid artery

**Supplemental figure 2:**

Transesophageal echo image, mid esophageal aortic valve long axis view at 135 degrees (systolic frame) showing a large mass (vegetation) attached to the bileaflet mechanical aortic valve complicated with the development of abscess formation and pseudoaneurysm. In real time note the systolic expansion of the pseudoaneurysm

**Supplemental figure 3:**

Intraoperative reconstruction of aortomitral curtain using bovine pericardial patch which was extended to the aortic root

**Supplemental figure 4:**

1. Intraoperative right femoral artery repair by patch angioplasty with great saphenous vein graft after endarterectomy and excision of embolic vegitations. B) Macroscopic appearance of the right femoral artery embolic vegetations
